# The Impact of Multimorbidity on All-Cause Mortality: A Longitudinal Study of 87,151 Thai Adults

**DOI:** 10.3389/ijph.2023.1606137

**Published:** 2023-10-10

**Authors:** Xiyu Feng, Haribondhu Sarma, Sam-Ang Seubsman, Adrian Sleigh, Matthew Kelly

**Affiliations:** ^1^ National Centre of Epidemiology and Population Health, Australian National University, Canberra, ACT, Australia; ^2^ School of Human Ecology, Sukhothai Thammathirat Open University, Pak Kret, Thailand

**Keywords:** all-cause mortality, lifestyle behaviour, multimorbidity, socio-demographics status, Thai Cohort Study

## Abstract

**Objectives:** To investigate associations between multimorbidity, socio-demographic and health behaviour factors, and their interactions (multimorbidity and these factors) with all-cause mortality among Thai adults.

**Methods:** Associations between multimorbidity (coexistence of two + chronic diseases) and mortality between 2005 and 2019 were investigated among Thai Cohort Study (TCS) participants (*n* = 87,151). Kaplan-Meier survival curves estimated and compared survival times. Multivariate Cox proportional hazards models examined associations between risk factors, and interactions between multimorbidity, these factors, and survival.

**Results:** 1,958 cohort members died between 2005 and 2019. The risk of death was 43% higher for multimorbid people. In multivariate Cox proportional hazard models, multimorbidity/number of chronic conditions, age, long sleep duration, smoking and drinking were all independent factors that increased mortality risk. Women, urbanizers, university education, over 20,000-baht personal monthly income and soybean products consumption lowered risk. The interactions between multimorbidity and these variables (except for female, urbanizers and soybeans intake) also had significant (*p* < 0.05) impact on all-cause mortality.

**Conclusion:** The results emphasise the importance of healthy lifestyle and reduced intake of alcohol and tobacco, in reducing premature mortality, especially when suffering from multimorbidity.

## Introduction

Multimorbidity refers to the co-existence of two or more chronic health conditions in a person [1, 2]. Moreover, the incidence of multimorbidity is positively correlated with increasing age [3]. While the proportion of the global population aged 60 or over was 9.8% in 2017, this proportion is projected to increase to 13.7% and 20.3% by 2030 and 2050 respectively. Older people face a variety of health problems, with multimorbidity being one of the most pressing [1, 2]. However, in recent years, a growing number of studies has found that multimorbidity has gradually ceased to be exclusive disease of the elderly and are becoming more prevalent among younger people [4, 5].

To date, approximately one-third of the global population worldwide suffers from multimorbidity, and this proportion is projected to rise by 2035 [2], which will pose a number of challenges to the health of the global population worldwide [1, 2]. The negative impacts of multimorbidity include an increased likelihood of disability, reduced quality of life and increased risk of death [3]. Evidence suggests that patients with multimorbidity are approximately 1.5 times more likely to die compared to those without multimorbidity [3]. In addition, studies in the UK [6] and China [7] have shown that all-cause mortality is higher in people with multimorbidity than in people without multimorbidity, and that the relative impact of an increased number of long-term chronic conditions on the increased risk of death is greater in younger and middle-age (around 35–50 years old) people [6]. Therefore, the management of multimorbidity has become a public health imperative.

Many factors which associate with multimorbidity such as age, gender, socioeconomic status (SES) and health behaviour have not been adequately studied in terms of their impact on the relationship between multimorbidity and mortality [8, 9]. For example, people with low SES [10] and those with risky individual health behaviours [11] may be more susceptible to multimorbidity. However, the impact of these factors on mortality, particularly for people with multimorbidity, has not been adequately studied. In particular, in low- and middle-income countries (LMICs) where multimorbidity is relatively under-researched, compared to high-income countries (HICs) [12].

Multimorbidity is a common condition in Southeast Asia, with a prevalence of about 16%. [13, 14], but there is relatively little research about this health burden in Southeast Asia [13, 14]. Thailand, a Southeast Asian country, is facing a health transition in disease patterns and causes of death. Non-communicable diseases (NCDs) such as diabetes and hypertension, are now the leading causes of death in Thailand [15]. However, little information is available on the interactions between the multimorbidity posed by these chronic conditions and the risk factors contributing to all-cause mortality. To better examine the relationship between multimorbidity and all-cause mortality in the Thai population, data from a long-term community-based cohort study in Thailand, the Thai Cohort Study (TCS), were used here. The TCS has studied more than 85,000 Thais living across the country since 2005, with a similar demographic profile of participants to the national population [15].

The aim of this study was to investigate whether multimorbidity and risk factors (socio-demographic and health behaviour factors) have a significant impact on survival in adults by independently analysing multimorbidity and risk factors, their interactions, and associations with mortality risk. It may be useful in order to inform health policy and health resource allocation in such a transitional context, and to design appropriate interventions to prevent or reduce the occurrence of multimorbidity and thereby reduce mortality.

## Methods

### The Study Population

The data used were from the longitudinal study of health transitions in Thailand, known as the Thai Cohort Study (TCS). Participants in the study were adults living throughout Thailand and attending Sukhothai Thammathirat Open University (STOU) [15, 16]. A comprehensive health questionnaire was mailed to all students in 2005 and over 80,000 people (age range: 15–87 years) responded. The questionnaire covered a wide range of topics, including socio-demographic data, indicators of health status, health behaviours and history of physician-diagnosed disorders [17].

### Mortality Data

TCS participants represented the median age of STOU’s student population and the total Thai population. They also represent the Thai population in terms of gender balance, ethnicity, religion, modest income, and geographical distribution. They are however on average more highly educated than Thais in general. Upon enrolment in the TCS, all participants were asked to provide a Citizenship Identification Number (CID), a unique 13-digit identification used for all government interactions [14]. The CID list of all TCS members was regularly checked against government death records maintained by the Thai Ministry of Interior [18]. For our study, we considered deaths among TCS members reported by the Ministry of Interior occurring between March 2005 and December 2019, for a total of 1,958 deaths. It was observed that the estimates of vital statistics kept in Thailand during our study period of high quality and completeness and were found to record 95% or more of the deaths [15, 18].

### The Definition of Multimorbidity

In our study, multimorbidity was defined as a person with two or more chronic conditions at the same time [1–3]. In the 2005 TCS baseline questionnaire, one of the questions was whether the respondent had been diagnosed by a doctor with any of the 18 diseases listed, with those with these illnesses reporting “yes” and those without them reporting “no” [15, 16]. Participants also recorded their weight and height, and BMI was calculated from these variables. Obesity was then defined as having a body mass index (BMI) greater than 25, with BMI greater than 25 classified as “yes” and BMI less than 25 classified as “no” [18]. Based on the specific cut-off for Asian populations, a BMI above 25 kg/m^2^ is defined as obese, rather than the WHO definition of over 30 kg/m^2^ as obese [19, 20]. Therefore, there were 18 disorders in our study. These chronic conditions were metabolic disorders: cardiometabolic conditions (diabetes, high cholesterol/high blood lipids, high blood pressure, ischemic (coronary) heart disease, cerebrovascular disease (stroke)), obesity, liver disease and kidney disease; endocrine disorders; goiter/thyroid abnormality; musculoskeletal disorders: arthritis; brain and emotional disorders: depression/anxiety and epilepsy; respiratory disorders: chronic bronchitis/lung disease and asthma; cancers: liver cancer, lung cancer, breast cancer and cancer of the digestive system. Multimorbidity was defined as the same individual having two or more of these 18 diseases being reported as “yes”. There was also created the other variable with more precise information on the number of chronic diseases named as the number of chronic conditions (0, 1, 2, 3, 4 or more chronic conditions, CC) [1].

### Independent Variables

The other factors were divided into two groups. The first group was socio-demographic factors, which included gender (male (reference) or female), age (years, ≤39 (reference) or 40–59 or ≥60), marital status (single (reference) or living with partner or married), geographic regions (Bangkok (reference) or Central or North or Northeast or East or South), life course residence (Rural-rural (lifelong ruralites, RR) (reference) or Rural-urban (urbanizers, RU) or Urban-rural (de-urbanizers, UR) or Urban-urban (urbanites, UU)), personal monthly income (baht, ≤7000(reference) or 7,001–10,000 or 10,001–20,000 or ≥20,001) and educational level (junior school (reference) or high school or diploma or university).

Among them, life course residence was categorized in terms of rural (R) or urban (U) residential residence, when aged 10–12 years old and again in 2005, creating four groups: lifelong ruralites (Rural-rural, RR), urbanizers (Rural-urban, RU), de-urbanizers (Urban-rural, UR) and urbanites (Urban-urban, UU) [16, 19, 21, 22]. As for personal monthly income, One US dollar were equivalent to around 25 Baht in 2005 [13].

The second group was health behaviours or personal lifestyles, which included dietary consumptions (deep fried food, instant food, soft drinks, soybean products: less than once a month (reference) or 1–3 times a month or 1–2 times a week or 3–6 times a week or daily or more; fruits and vegetables: 0–4 (reference) or 5–9 or 10–14 or ≥15). The grouping of intake of fruits and vegetables would be consistent with that of previous articles [16, 19, 21, 22].

As for the active status, there were four groups and the first one is weekly exercise. Information on weekly exercise was obtained by asking: “during a typical week (7-day period) how many times on average do you do each of these physical activities”: “vigorous exercise over 20 min,” “moderate exercise over 20 min” and “walking for at least 10 min.” In this study, however, we used these three indictors to calculate the adjusted exercise measure (number of sessions): “2 × vigorous exercise +1 × moderate exercise +1 × number of walks per week” to weight weekly activity intensity. This adjusted exercise measure was based on the International Physical Activity Questionnaire and the Australian Active Survey (IPAQ and AAS) [21, 22], since the question of weekly exercise is a sessions-based measure of physical activity, similar to the sessions component of the is similar to IPAQ and AAS, and many studies in TCS used this measure to weight weekly exercise [19, 21, 22]. Finally, weekly exercise was recoded as “0–7 sessions” (reference) or “8–14 sessions” or “more than 15 sessions,” which was consistent with the grouping of previous articles [21, 22]. The other three groups were the frequency of housework (1–3 times a month (reference) or 1–2 times a week or 3–4 times a week or every day or more), sleeping time (hours/day: ≤6 h or 7–8 h (reference) or ≥9 h) and sedentary time (hours/day: ≤7 h (reference) or 8–12 h or ≥13 h).

In terms of smoking, there were two measures on the questionnaire, the first being never smoking (reference) and the second being smoking, including former smokers who have now quit and those who have always smoked. As for drinking status, information was obtained by asking participants and through their self-report: “Have you ever drunk alcohol”: “Occasional social drinker,” “‘No, never” (reference), “Current regular drinker” or “Used to drink before, now stopped” (ex-drinker).

### Statistical Analysis

The main variable of interest was whether the cohort member died between March 2005 and December 2019. A stratified analysis has been performed for gender and age (Table 3). We described the distribution of deaths by individual characteristics of cohort members: participants, multimorbidity, number of chronic conditions (5 categories), age groups (3 categories), marital status (3 categories), regions area of residence (geographic regions, 6 categories), life course residence (urban or rural resident at age 10 to 12 and in 2005), education level (4 categories) and monthly income (4 categories, baht, US$1 was equal to 25 baht in 2005). We used Stata 16.0 to tackle the analysis.

Kaplan Meier survival curves display explain multimorbidity or not/different number of chronic conditions by baseline in 2005. End-point events were all-cause deaths until 2019. We used the log-rank test to test the statistical probability of observed difference in survival patterns according to having multimorbidity or not/different number of chronic conditions. Univariate and multivariate Cox proportional hazards models were used to examine the association between risk factors (including multimorbidity and the number of chronic conditions) and survival [23]. Multivariate Cox proportional hazards models were also used to evaluate the association between the interaction between multimorbidity and these independent variables, and survival. Hazard ratios (HR) and 95% confidence intervals (95% CI) were generated as the results [23]. For each set of associations, we adjusted other factors in Table 2 for confounders. For any missing data, our study assumed that they were missing at random. For all analyses, p for trend (p-trend) were used to test the linear trend and *p* values <0.05 were considered statistically significant. All statistical analyses were performed using Stata 16.0.

### Ethical Approval

All participants obtained informed written consent and were informed that they could withdraw or not participate in the study without affecting their academic progress.

The Thai Cohort Study (TCS) has been approved by the ethic committee of the Sukhothai Thammathirat Open University Research and Development Institute (protocol number: 0522/10). For this sub-sample analysis, we also got approval from Human Research Ethics Committee of the Australian National University (protocol number: 2004/344, 2009/570, 2021/796).

## Results

### Cohort Characteristics by Survival Status at the Baseline

A total of 1,958 members of the Thai Cohort Study died between 2005 and 2019. The distribution of these deaths across socioeconomic and demographic groups is reported in Supplementary Table S1 in Supplementary File S1. Among deceased cohort members, males accounted for approximately 70% of the total deaths. The cumulative mortality in the groups of having multimorbidity and/or more than 4 chronic conditions (CC) were higher than other groups. Rural groups accounted for a higher proportion of deaths, particularly those who had lived in rural areas since childhood. In addition, groups under 39 years of age, high school graduates and those earning less than 7,000 baht per month were more likely to be in the death group.

### Survival Analysis

**FIGURE 1:**
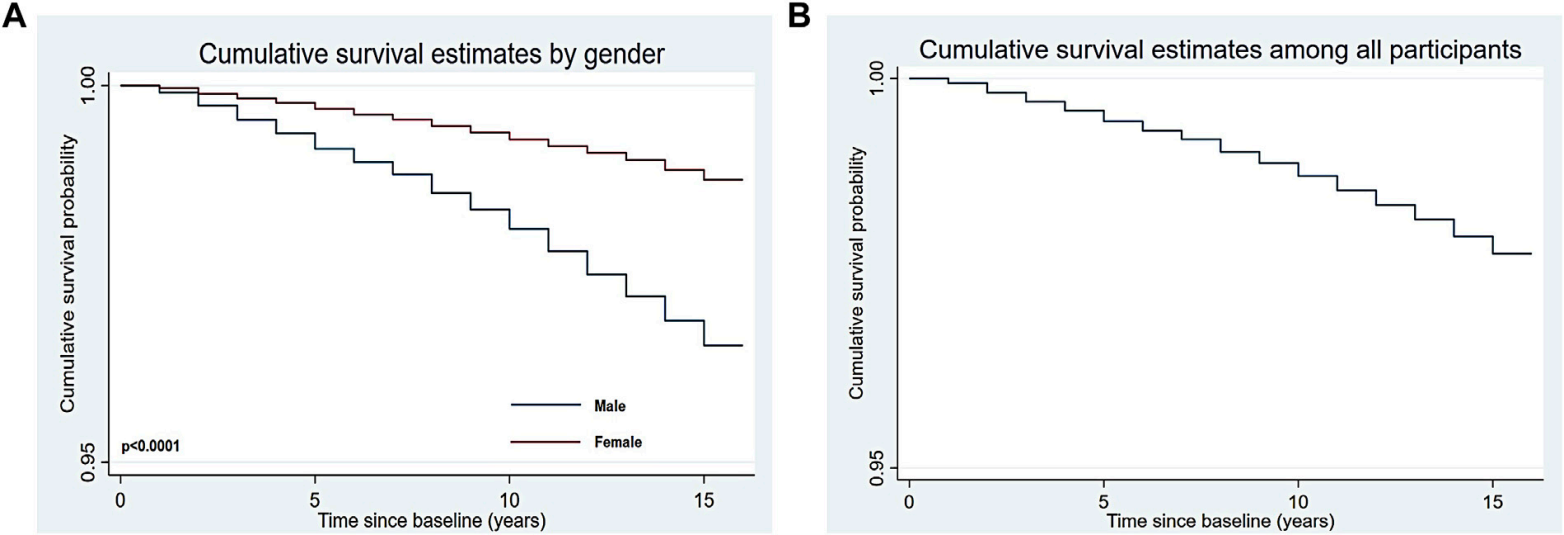
Kaplan-Meier estimated curves for cumulative survival (**(A)** for male and female; **(B)** for all participants) (Thailand, 2005 for baseline characteristics and 2019 for all-cause mortality).

**FIGURE 2:**
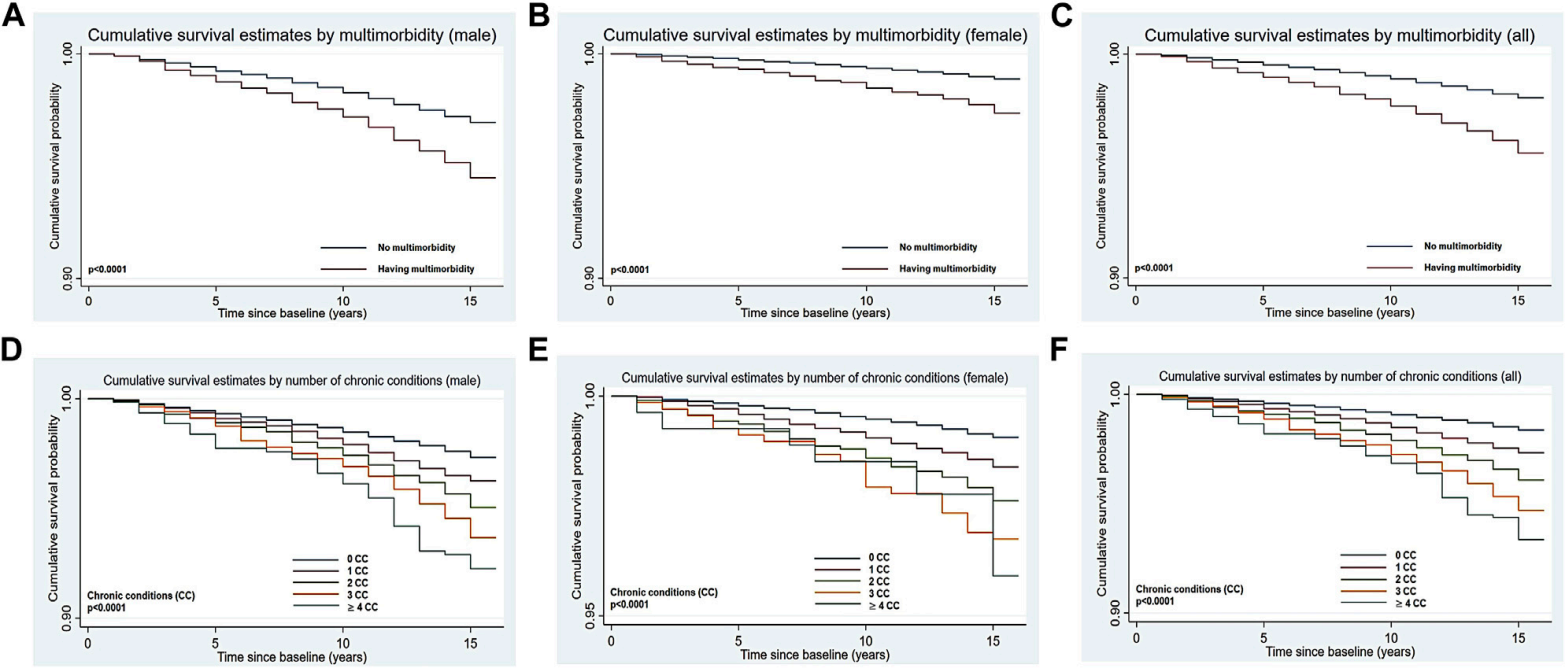
Kaplan-Meier estimated curves for cumulative survival by multimorbidity (**(A)** for male; **(B)** for female; **(C)** for all) or number of chronic conditions (**(D)** for male; **(E)** for female; **(F)** for all), and gender (Thailand, 2005 for baseline characteristics and 2019 for all-cause mortality).

As for the Kaplan-Meier analysis (see Figure 1A), the 15 years cumulative survival probabilities in male participants were 96.55% (95% CI: 96.37%–96.73%) and for female members were 98.75% (95% CI: 98.65%–98.85%) (*p* < 0.0001). Moreover, the cumulative survival probabilities of all members at 15 years were 97.75% (95% CI: 97.65%–97.85%) (see Figure 1B). Significant differences (all *p*-value<0.0001) in cumulative survival time were shown among multimorbid patients and others (see Figures 2A–C). The 15 years cumulative survival probabilities in males with multimorbidity were 94.48% (95% CI: 93.89%–95.02%), female with multimorbidity were 97.35% (95% CI: 96.80%–97.81%) and all people with multimorbidity were 95.58% (95% CI: 95.16%–95.96%).

In the analysis of multimorbidity and other risk factors of mortality (see Table 1 and Supplementary Table S2 in Supplementary File S1), people with multimorbidity (compared to others without multimorbidity; hazard ratio (HR): 2.29, 95% CI: 2.06–2.54, adjusted hazard ratio (AHR):1.43, 95% CI: 1.26–1.62), older age (compared to less than 39 years old people; 40–59 years old: HR: 2.84, 95% CI: 2.58–3.13, AHR: 2.33, 95% CI: 2.03–2.67; over 60 years old: HR: 18.47, 95% CI: 14.88–22.93, AHR: 11.46, 95% CI: 8.58–15.30), sleeping over 9 h (compared to sleeping time between 7–8 h; HR: 1.19, 95% CI: 1.04–1.36, AHR: 1.25, 95% CI: 1.08–1.44), smoking (compared to non-smokers; smoking: HR: 2.58, 95% CI: 2.36–2.82, AHR: 1.41, 95% CI:1.24–1.60), drinking (compared to non-drinkers; ex-drinker: HR: 0.81, 95% CI: 0.72–0.91, AHR: 1.17, 95% CI: 1.01–1.35; social drinker: HR: 2.22, 95% CI: 1.90–2.59, AHR: 1.37, 95% CI: 1.15–1.63; regular drinker: HR: 2.02, 95% CI: 1.78–2.29, AHR: 1.44, 95% CI: 1.25–1.67) independently predicted much poorer cumulative survival. Moreover, the more frequently alcohol was consumed, the worse the cumulative survival probability (p-trend = 0.0160).

**TABLE 1:** Multivariate adjusted hazard ratios according to multimorbidity, the number of chronic conditions (CC) and risk factors (Thailand, 2005 for baseline characteristics and 2019 for all-cause mortality).

Participants							
	Male		Female		Total		
	AHR*	(95% CI)	AHR*	(95% CI)	AHR*	(95% CI)	
Two or more chronic conditions							
Multimorbidity							
No	1.00		1.00		1.00		
Yes	**1.36**	**(1.18, 1.57)**	**1.76**	**(1.38, 2.25)**	**1.43**	**(1.26, 1.62)**	
Number of chronic conditions (CC)							
0 CC	1.00		1.00		1.00		
1 CC	**1.23**	**(1.07, 1.42)**	**1.57**	**(1.29, 1.92)**	**1.34**	**(1.20, 1.51)**	
2 CC	**1.40**	**(1.16, 1.68)**	**1.87**	**(1.38, 2.53)**	**1.52**	**(1.30, 1.78)**	
3 CC	**1.74**	**(1.35, 2.25)**	**2.77**	**(1.74, 4.42)**	**1.92**	**(1.54, 2.41)**	
≥4 CC	**1.69**	**(1.19, 2.41)**	**3.00**	**(1.52, 5.90)**	**1.87**	**(1.37, 2.55)**	
p-trend	**0.0097**		**0.0022**		**0.0098**		
Socio-demographic factors							
Gender	1.00		**0.53**	**(0.46, 0.61)**			
Age (years)							
≤39 years	1.00		1.00		1.00		
40–59 years	**2.26**	**(1.92, 2.67)**	**2.41**	**(1.86, 3.13)**	**2.33**	**(2.03, 2.67)**	
≥60 years	**11.96**	**(8.80, 16.26)**	**8.29**	**(2.59, 26.56)**	**11.46**	**(8.58, 15.30)**	
p-trend	0.1931		0.1400		0.1891		
Life course residence**							
Rural-rural (RR)	1.00		1.00		1.00		
Rural-urban (RU)	0.86	(0.74, 1.00)	**0.79**	**(0.62, 1.00)**	**0.84**	**(0.74, 0.95)**	
Urban-rural (UR)	1.26	(0.98, 1.62)	0.98	(0.64, 1.51)	1.19	(0.95, 1.48)	
Urban-urban (UU)	0.94	(0.79, 1.13)	1.17	(0.93, 1.49)	1.04	(0.91, 1.20)	
Education level							
Junior school	1.00		1.00		1.00		
High school	**0.76**	**(0.60, 0.96)**	1.02	(0.59, 1.76)	0.81	(0.66, 1.00)	
Diploma	0.81	(0.64, 1.04)	0.92	(0.53, 1.60)	0.83	(0.66, 1.03)	
University	**0.72**	**(0.56, 0.93)**	0.64	(0.36, 1.12)	**0.68**	**(0.55, 0.86)**	
p-trend	0.1770		0.1315		0.0765		
Personal monthly income (baht)							
≤ 7,000 baht	1.00		1.00		1.00		
7,001–10000 baht	1.03	(0.87, 1.22)	1.06	(0.83, 1.36)	1.05	(0.91, 1.21)	
10,001–20000 baht	**0.82**	**(0.68, 0.98)**	1.20	(0.93, 1.55)	0.92	(0.80, 1.07)	
≥ 20,001 baht	**0.73**	**(0.59, 0.92)**	0.99	(0.69, 1.42)	**0.80**	**(0.66, 0.97)**	
p-trend	0.0966		0.7921		0.0598		
Health behaviours/Personal lifestyles							
Diet consumptions							
Soybean products							
Never	1.00		1.00		1.00		
1–3 times/month	0.89	(0.74, 1.07)	**0.72**	**(0.52, 0.99)**	**0.85**	**(0.72, 0.99)**	
1–2 times/week	0.97	(0.81, 1.16)	1.02	(0.76, 1.37)	0.99	(0.85, 1.16)	
3–6 times/week	0.94	(0.77, 1.15)	1.07	(0.79, 1.44)	0.99	(0.84, 1.16)	
Daily or more	1.03	(0.80, 1.32)	0.97	(0.69, 1.35)	1.00	(0.83, 1.22)	
p-trend	0.5980		0.5815		0.5752		
Activity status							
Housework							
Never	1.00		1.00		1.00		
1–3 times/month	1.03	(0.79, 1.34)	0.80	(0.49, 1.28)	0.98	(0.78, 1.24)	
Once or twice/week	1.03	(0.81, 1.31)	0.73	(0.48, 1.10)	0.97	(0.79, 1.20)	
3–4 times/week	1.01	(0.77, 1.33)	0.71	(0.45, 1.12)	0.95	(0.75, 1.20)	
Every day or more	1.21	(0.96, 1.53)	0.69	(0.46, 1.04)	1.07	(0.87, 1.31)	
p-trend	0.1645		**0.0450**		0.5318		
Sleeping time (hours)							
≤6 h	1.06	(0.93, 1.21)	0.99	(0.81, 1.21)	1.04	(0.94, 1.17)	
7–8 h	1.00		1.00		1.00		
≥9 h	**1.25**	(1.04, 1.49)	1.24	(0.96, 1.60)	**1.25**	**(1.08, 1.44)**	
p-trend	0.4810		0.3108		0.4285		
Smoking and Drinking status							
Smoking							
Never smoking	1.00		1.00		1.00		
Smoking	**1.45**	**(1.26, 1.66)**	1.29	(0.92, 1.80)	**1.41**	**(1.24, 1.60)**	
Drinking							
Never	1.00		1.00		1.00		
Ex-drinker	**1.26**	**(1.02, 1.57)**	1.11	(0.91, 1.35)	**1.17**	**(1.01, 1.35)**	
Occasional or social	**1.37**	**(1.15, 1.65)**	1.79	(0.83, 3.86)	**1.37**	**(1.15, 1.63)**	
Current regular	**1.41**	**(1.19, 1.67)**	**1.57**	**(1.16, 2.13)**	**1.44**	**(1.25, 1.67)**	
p-trend	0.0627		0.1762		**0.0160**		

AHR*: Multivariate adjusted Hazard ratios from Cox proportional hazard models controlling for other socio-demographic factors and personal lifestyle elements (variables in Supplementary Table S2 in Supplementary File S1). AHR bolding: The results of AHR were statistical significance (*p* < 0.05). p-trend bolding: The results of p-trend were statistical significance (p for trend <0.05). Life course residence**: Life course residence was categorized in terms of rural (R) or urban (U) residential residence, when aged 10–12 years old and again in 2005, creating four groups: lifelong ruralites (Rural-rural, RR), urbanizers (Rural-urban, RU), de-urbanizers (Urban-rural, UR) and urbanites (Urban-urban, UU) [16, 19, 21, 22].

On the other hand (see Table 1 and Supplementary Table S2 in Supplementary File S1), female participants (compared to men; HR: 0.36, 95% CI: 0.33–0.40, AHR: 0.53, 95% CI: 0.46–0.61), people who were urbanised (Rural-urban (RU), HR: 0.91, 95% CI: 0.81–1.01, AHR: 0.84, 95% CI: 0.74–0.95), compared to participants who were lifelong ruralites (Rural-rural, RR), completion of university degree (compared to members with primary school; HR: 0.39, 95% CI: 0.32–0.47, AHR: 0.68, 95% CI: 0.55–0.86), monthly personal income over 20,000 baht (compared to members with less 7,000 baht per month; HR: 1.64, 95% CI: 1.42–1.88, AHR: 0.80, 95% CI: 0.66–0.97) and consuming soybean products 1–3 times per month (compared to people who never eating soybean products; HR: 0.80, 95% CI: 0.69–0.92, AHR: 0.85, 95% CI: 0.72–0.99) showed better cumulative survival. As for activity status, the risk of death declined more for women who did housework more frequently, with a linear trend (p-trend = 0.0450).

Furthermore, with regard to the number of chronic conditions (CC), in the comparison with people without chronic conditions, the higher the number of conditions, the higher the risk of death among these participants (one CC: HR: 1.64, 95% CI: 1.48–1.82; two CC: HR: 2.41, 95% CI: 2.11–2.76; three CC: HR: 3.29, 95% CI: 2.70–4.02; over four CC: HR: 4.15, 95% CI: 3.19–5.40) in crude analysis, with a linear trend (p-trend = 0.0001) (Supplementary Table S2 in Supplementary File S1). Such a trend would be also unchanged after controlling for socio-demographic and behavioural factors (see Table 1), compared to individuals with no chronic conditions, having one chronic disease may increase the risk of death by 34%, while having two diseases may increase the risk by 52%, having three diseases would near double the risk, and four and more diseases would be an 87% risk, which would be close to a linear increase (p-trend = 0.0098).

The more the number of chronic diseases people suffered from, the lower the probability of survival they were (*p* < 0.0001) (see Figures 2D–F). The 15 years cumulative survival probability was 1.03% lower for all people with one disease than for people without chronic diseases, 2.25% lower for two diseases, 4.29% lower for three diseases, and 5.00% lower for four or more diseases, which was a significant linear relationship (p-trend = 0.0011).

**FIGURE 3:**
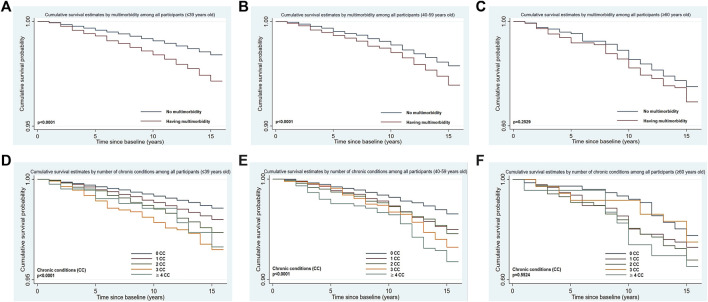
Kaplan-Meier estimated curves for cumulative survival by multimorbidity (**(A)** for ≤ 39 years old; **(B)** for 40-59 years old; **(C)** for ≥ 60 years old) or number of chronic conditions (**(D)** for ≤ 39 years old; **(E)** for 40-59 years old; **(F)** for ≥ 60 years old), and age (Thailand, 2005 for baseline characteristics and 2019 for all-cause mortality).

For all people (see Figures 3A–C), the 15 years survival probability of multimorbidity decreased when the age increased (less than 39 years old: 97.15% (95% CI: 96.72%–97.53%) vs. 40–59 years old: 93.92% (95% CI: 93.11%–94.64%) vs. over 60 years old: 69.18% (95% CI: 61.00%–75.98%). The risk of death for multimorbidity was significantly increased when people got older but there was no linear relationship between them (see Table 2). In terms of the number of chronic conditions (see Figures 3D–F; Table 2), a statistically significant difference was found between number of chronic conditions and overall survival time in different age groups (*p* < 0.0001, except over 60 years old group (*p* = 0.5524)). The risk of death increased with age for the same number of diseases, but there was no significant linear relationship. In the group of 40–59 years old, the risk of death increased with increased number of chronic conditions, with the significant linear relationship (p-trend = 0.0070).

**TABLE 2:** Hazard ratios according to number of multimorbidity or chronic conditions, and age (Thailand, 2005 for baseline characteristics and 2019 for all-cause mortality).

Participants									
	Univariate hazard ratios (HRs) (95% CI)				Multivariate adjusted hazard ratios (AHRs*) (95% CI)				
	Age groups				Age groups				
	≤39 years old	40–59 years old	≥60 years old	p-trend	≤39 years old	40–59 years old	≥60 years old	p-trend	
Multimorbidity									
No	1.00	1.00	1.00	NA	1.00	1.00	1.00	NA
Yes	1.80 (1.54, 2.10)	1.45 (1.23, 1.71)	1.27 (0.84, 1.93)	0.1302	**1.49 (1.25, 1.77)**	**1.37 (1.14, 1.66)**	**3.02 (1.43, 6.44)**	0.3545
Number of chronic conditions (CC)									
0 CC	1.00	1.00	1.00	NA	1.00	1.00	1.00	NA
1 CC	1.40 (1.24, 1.58)	1.46 (1.19, 1.79)	1.3 (0.70, 2.39)	0.5618	**1.26 (1.09, 1.44)**	**1.59 (1.25, 2.01)**	1.19 (0.41, 3.49)	0.8868
2 CC	1.86 (1.54, 2.25)	1.58 (1.25, 1.99)	1.56 (0.86, 2.86)	0.3090	**1.50 (1.21, 1.85)**	**1.60 (1.22, 2.11)**	**3.76 (1.35, 10.52)**	0.2868
3 CC	2.47 (1.79, 3.41)	1.98 (1.47, 2.67)	1.13 (0.50, 2.54)	0.0844	**2.09 (1.48, 2.95)**	**2.06 (1.47, 2.89)**	**3.54 (1.04, 12.11)**	0.3217
≥4 CC	2.37 (1.39, 4.01)	2.43 (1.70, 3.48)	1.73 (0.76, 4.00)	0.3690	1.63 (0.87, 3.06)	**2.25 (1.48, 3.41)**	2.25 (0.60, 8.47)	0.3566
p-trend	**0.0096**	**0.0018**	0.2082	NA	0.0980	**0.0070**	0.2885	NA

NA: Not available. AHRs*: Multivariate adjusted Hazard ratios from Cox proportional hazard models controlling for other socio-demographic factors and personal lifestyle elements (variables in Supplementary Table S2 in Supplementary File S1). AHRs bolding: The results of Multivariate hazard ratios (HR) were statistical significance (*p* < 0.05). p-trend bolding: The results of p-trend were statistical significance (p for trend <0.05).

As for the impact of interactions between multimorbidity and risk factors on all-cause mortality (see Table 3), in the adjusted model (controlling socio-demographic and behavioural factors), females with multimorbidity had 6% lower risk of death than males without multimorbidity, but this result was not statistically significant. There is a linear increase (p-trend = 0.0413) in the risk of death with age for people with multimorbidity in comparison to people younger than 39 years who do not have multimorbidity. In the terms of socio-economic status (SES), higher-income and better-educated (high SES) multimorbid people have a lower risk of death (AHR: 0.49, 95% CI: 0.32–0.75) than healthy people with low SES. Members with multimorbidity have a significantly (all *p* < 0.05) increased risk of death, regardless of the amount of sleep they get, compared to normal participants (without multimorbidity) who get a normal amount of sleep (7–8 h).

**TABLE 3:** Multivariate adjusted hazard ratios according to the interaction between multimorbidity and risk factors (Thailand, 2005 for baseline characteristics and 2019 for all-cause mortality).

**Participants**	Male		Female		Total	
	AHR*	(95% CI)	AHR*	(95% CI)	AHR*	(95% CI)
Socio-demographic factors						
Multimorbidity*gender (References: no multimorbidity*male)						
having multimorbidity	**1.32**	**(1.14, 1.52)**	0.94	(0.74, 1.19)		
Multimorbidity*age (years) (References: no multimorbidity*≤39 years)						
Having multimorbidity*≤39 years	**1.40**	**(1.13, 1.73)**	**1.83**	**(1.34, 2.51)**	**1.51**	**(1.27, 1.80)**
Having multimorbidity*40–59 years	**3.11**	**(2.52, 3.84)**	**4.06**	**(2.78, 5.95)**	**3.27**	**(2.72, 3.93)**
Having multimorbidity*≥60 years	**17.03**	**(11.59, 25.03)**	**26.53**	**(8.20, 85.81)**	**17.41**	**(12.14, 24.97)**
p-trend	**0.0421**		0.0837		**0.0413**	
Socio-economic status (SES)						
Multimorbidity*Personal monthly income (baht)*Education level (References: no multimorbidity*≤ 7,000 baht*junior school)						
Having multimorbidity*≥20,001 baht*junior school	0.70	(0.32, 1.53)	1.91	(0.38, 9.73)	0.81	(0.40, 1.62)
Having multimorbidity*≥20,001 baht*high school	**0.39**	**(0.22, 0.69)**	0.82	(0.23, 2.97)	**0.43**	**(0.26, 0.72)**
Having multimorbidity*≥20,001 baht*diploma	0.58	(0.33, 1.02)	0.92	(0.26, 3.33)	0.61	(0.37, 1.03)
Having multimorbidity*≥20,001 baht*university	**0.50**	**(0.31, 0.79)**	0.46	(0.14, 1.54)	**0.49**	**(0.32, 0.75)**
p-trend	0.4263		**0.0253**		0.1948	
Health behaviours/Personal lifestyles						
Activity status						
Multimorbidity*Sleeping time (hours) (References: no multimorbidity*7–8 h)						
Having multimorbidity*≤6 h	**1.48**	**(1.20, 1.84)**	**1.69**	**(1.14, 2.51)**	**1.51**	**(1.25, 1.83)**
Having multimorbidity*7–8 h	**1.25**	**(1.02, 1.53)**	**1.61**	**(1.14, 2.28)**	**1.31**	**(1.10, 1.57)**
Having multimorbidity*≥ 9 h	**1.81**	**(1.31, 2.52)**	**2.82**	**(1.64, 4.86)**	**2.01**	**(1.52, 2.66)**
p-trend	0.6013		0.3710		0.5122	
Smoking and drinking status						
Multimorbidity*Smoking (References: no multimorbidity*never smoking)						
Having multimorbidity*never smoking	**1.57**	**(1.21, 2.02)**	**1.75**	**(1.35, 2.28)**	**1.68**	**(1.40, 2.01)**
Having multimorbidity*smoking	**2.12**	**(1.73, 2.58)**	**2.39**	**(1.29, 4.42)**	**2.05**	**(1.71, 2.46)**
Multimorbidity*Drinking (References: no multimorbidity*never)						
Having multimorbidity*never	**1.36**	**(1.13, 1.64)**	**1.81**	**(1.28, 2.58)**	**1.43**	**(1.21, 1.68)**
Having multimorbidity*ex-drinker	**2.19**	**(1.48, 3.24)**	**1.86**	**(1.26, 2.75)**	**2.03**	**(1.54, 2.66)**
Having multimorbidity* occasional or social	**1.70**	**(1.25, 2.32)**	0.00		**1.65**	**(1.21, 2.24)**
Having multimorbidity*current regular	**1.87**	**(1.41, 2.49)**	**3.33**	**(1.89, 5.89)**	**2.02**	**(1.57, 2.60)**
p-trend	0.7108		0.7443		0.3898	

AHRs*: Multivariate adjusted Hazard ratios from Cox proportional hazard models controlling for other socio-demographic factors and personal lifestyle elements (variables in Supplementary Table S2 in Supplementary File S1). AHRs bolding: The results of Multivariate hazard ratios (HR) were statistical significance (*p* < 0.05). p-trend bolding: The results of p-trend were statistical significance (p for trend <0.05).

However, it is worth noting that multimorbid patients with 7–8 h (AHR: 1.31, 95% CI: 1.10–1.51) of sleep had a slightly lower risk of death than those with abnormal sleep schedules (less than 6 h: AHR: 1.51, 95% CI: 1.25–1.83; more than 9 h: AHR: 2.01, 95% CI: 1.52–2.66). Individuals who smoked and had multimorbidity were 1.05 times more likely to be at risk of death than those who did not smoke and did not have multimorbidity. Participants with multimorbidity have an increased risk of death whether they drink alcohol or not, compared to healthy people who never drink alcohol. While alcohol consumption increased the risk of death even more. The risk of death increases 43% for the multimorbid individuals that never drinks alcohol and 102% for the multimorbid members that drinks alcohol regularly (see Table 3).

## Discussion

Our findings suggest that multimorbidity as an independent risk factor reduces the chances of survival and increases the risk of death in this study, over 15 years of follow-up. The higher the number of diseases suffered by participants, the lower the chances of survival. Across age groups, the risk of death for those with multimorbidity increased and the chance of survival decreases with increasing age. Compared to young people without multimorbidity, the older the person is, the higher the risk of death if they have multimorbidity at the same time. In addition, male sex, sleeping longer, smoking and drinking alcohol were risk factors for death, while female sex, urbanizing, university graduate status, high personal monthly income (more than 20,000 baht) and consuming soybean products were protective factors. Furthermore, when people suffering from multimorbidity, also have abnormal sleeping time, smoke and drink alcohol the risk of death further increases, whereas high socio-economic status (SES) (university graduate status and more than 20,000 baht monthly) could reduce the risk of death.

Age is the most important factor driving multimorbidity. Like the results of other studies [8], the older the person was the higher their risk of death if also having multimorbidity. This may be related to the fact that as age increased, the chances of developing multimorbidity increased, thus raising the risk of death, and that age itself is an independent risk factor for death [1, 2]. However, the aging of Thai society is getting serious. In 2020, about one-fifth of the population were over 60 years old, and this proportion will increase to about 30% in 2030 [24]. Therefore, how to delay the development of age-related health conditions in the society and improve the health of the elderly is particularly important for Thailand [13].

In terms of gender, we found that women participants appear to be more likely to survive than men regardless of these females with or without multimorbidity, which is similar to the findings of a European study [1]. This may be due to genetic differences between males and females [25], unmeasured confounders such as differences in health-seeking behaviours between men and women [25], and males would be susceptible to diseases with higher mortality rates, whereas females are more likely to suffer from chronic diseases with higher rates of disability [26], all of which may make females less exposed to the risk of death. Therefore, incorporating gender perspectives into health interventions is essential. It is worth noting, however, that Thai women in general may experience more health inequalities because their social welfare indicators still lag behind those of Thai men [27].

The risk of death was also lower among those with university-level education, and/or those with a monthly income of over 20,000 baht, which is consistent with the findings of a study examining the relationship between socio-economic inequality and mortality, that is, the risk of death was lower among those with high income and/or high education compared to people with low income and/or low education [28]. People who move from rural to urban areas (urbanizers) also have a lower risk of death compared to those who are lifelong living in rural places, and such results are similar to those of a US study [29]. Also, high-income and highly educated people, namely, those with high SES who suffer from multimorbidity, also have a lower risk of death than healthy people with low SES. This outcome is similar to the findings of the Danish [30] and Canadian studies [31]. As this illustrates, people of higher SES have a lower risk of mortality since they have more financial and material resources to maintain a healthy life, to reduce the incidence of accidents, and to prevent and treat diseases more effectively [28–31].

People who sleep more than 9 h per day also had an increased risk of death compared to those who had a standard sleep duration (6–8 h), and such results were consistent with the findings of a systematic review on sleep duration and mortality [32]. In addition, for people with multimorbidity, abnormal sleep duration can also cause an increased risk of death, and they have a greater risk of death than people with multimorbidity who have a normal sleep duration. Such results are similar to the findings of a Chinese study on sleep duration and multimorbidity [33]. Thus, excessive or short sleep duration may increase the risk of death, which may be due to the fact that sleeping too long or too short may cause damage to the endocrine system and metabolism, which may lead to a range of diseases, including hypertension and diabetes, which may cause or exacerbate multimorbidity, then leading to death [32, 33]. It is also important to note that not only does sleep duration have an impact on chronic conditions, but chronic diseases also affect sleep duration because patients with multimorbidity may also have abnormal sleep duration due to their conditions such as pain and weakness [32, 33]. Thus, multimorbidity and sleep time abnormalities can act either independently or interact to increase the risk of death [32, 33].

Although we could not find the significant results of the interaction between the intake of soybeans and multimorbidity, the consumption of soybean products, as the independent variable, could decrease the risk of death in our study, which is consistent with a study in China [34]. This is because soy consumption lowers cholesterol and thus reduces the risk of cardiovascular diseases (CVDs), which is the main cause of disability-adjusted life years (DALYs) lost worldwide [34]. Furthermore, similarly, while in our study, the interaction between multimorbidity and frequency of housework resulted in a statistically insignificant effect on all-cause mortality. However, when frequency of housework was used as an independent variable, for women, more frequent housework was inversely linear associated with the risk of death, which is similar to the findings of a study in China that found that housework reduced the risk of death from diseases related to cancer [35], In our findings, smoking [2, 3, 36] and alcohol consumption [2, 7, 36] increased the risk of death in the population with or without multimorbidity, which is consistent with most studies [2, 3, 7, 36]. The increased risk of death due to smoking and alcohol consumption may be due to cardiovascular diseases and related cancers, such as liver and lung cancers [34], which may predispose to or aggravate chronic conditions [36, 37].

### Strengths and Limitations

This study is a 15 years study and with over 80,000 participants and 1,958 death cases, the sample size is very large and rich. It is also the first study to use the TCS to examine multimorbidity (and to divide it into different numbers of diseases) and mortality, and their associated risk factors. The other strength is also that it uses official Thai government mortality data, which has a high degree of veracity [13].

However, the study also has limitations. The age distribution of cohort members was generally younger and cohort members were more highly educated than the general population. In addition, all variables except mortality status are self-reported and therefore subject to some measurement error and or recall bias [13, 23].

Since the cause of death was not presented, this study used all-cause mortality as the primary outcome, which means that it would have some limitations in connecting multimorbidity to risk of cause-specific of death. However, this measure would have some advantages in terms of begin able to see the big picture risks associated with multimorbidity, which has the potential to be diluted through breaking up the deaths into multiple specific causes. Future analysis in this cohort study will focus on causes of death.

### Further Research

Therefore, in further studies, the focus should be on investigating the causes of death in the cohort study and will begin to establish potential causal relationships between identified risk factors and causes of death, particularly in relation to multimorbidity [15, 23]. Additionally, more attention should also be paid to the issue of ageing in Thailand and to increasing research into the prevention and treatment of multimorbidity in collaboration with the local Thai government [13].

### Conclusion

In the study, which lasted 15 years, multimorbidity and a higher number of co-existing diseases, age, long sleep duration, smoking and alcohol consumption were all independent factors that increased the risk of death. On the other hand, women, moving to urban places, high monthly income, university degrees and intake of soybean food may have reduced their risk of death. Moreover, in addition to gender, life course residence and the consumption of soybean products, the interaction between multimorbidity and these variables would also have a significant effect on all-cause mortality. It is hoped that these findings would help people to take their health seriously and that the authorities would strengthen the control and intervention of multimorbidity to improve the quality of life of people.
