# Supplementary material for: The Impact of Multimorbidity on All-Cause Mortality: A Longitudinal Study of 87,151 Thai Adults
Source: Int J Public Health. 2023 Oct 10;68:1606137. doi: 10.3389/ijph.2023.1606137 (PMC10594150; doi:10.3389/ijph.2023.1606137)
Supplement: Supplementary file 1 [file DataSheet1.docx]

**International Journal of Public Health**

**The impact of multimorbidity on all-cause mortality: A longitudinal study of 87151 Thai adults**

**Supplementary Table 1: Cohort characteristics by survival status (Thailand, 2005 for baseline characteristics and 2019 for all-cause mortality)**.

| **Factors** | **Male** |  |  |  | **Female** |  |  |  | **Total** |  |  |  |
| --- | --- | --- | --- | --- | --- | --- | --- | --- | --- | --- | --- | --- |
|  | **Alive** | **Dead** | **All** | **CMR per 1000*** | **Alive** | **Dead** | **All** | **CMR per 1000*** | **Alive** | **Dead** | **All** | **CMR per 1000*** |
|  | **N (%)** | **N (%)** | **N (%)** |  | **N (%)** | **N (%)** | **N (%)** |  | **N (%)** | **N (%)** | **N (%)** |  |
| **Participants** | 38123 (44.75) | 1362 (69.56) | 39,485 (45.28) | 34.50 | 47,062 (55.25) | 596 (30.44) | 47,658 (54.69) | 12.51 | 85,185 (100.00) | 1,958 (100.00) | 87,143 (100.00) | 22.47 |
| **p-value** |  |  |  |  |  |  | p<0.0001 |  |  |  |  |  |
| **Multimorbidity** |  |  |  |  |  |  |  |  |  |  |  |  |
| **No** | 32113 (84.24) | 1011 (74.23) | 33124 (83.89) | 30.52 | 43237 (91.87) | 492 (82.55) | 43729 (91.76) | 11.25 | 75350 (88.45) | 1503 (76.76) | 76853 (88.20) | 19.56 |
| **Yes** | 6010 (15.76) | 351 (25.77) | 6361 (16.11) | 55.18 | 3825 (8.13) | 104 (17.45) | 3929 (8.24) | 26.47 | 9835 (11.55) | 455 (23.24) | 10290 (11.81) | 44.22 |
| **p-value** |  |  |  | p<0.0001 |  |  |  | p<0.0001 |  |  |  | p<0.0001 |
| **Number of chronic conditions (CC)** | |  |  |  |  |  |  |  |  |  |  |  |
| **0 CC** | 20725 (54.36) | 569 (41.78) | 21294 (53.93) | 26.72 | 31461 (66.85) | 299 (50.17) | 31760 (66.64) | 9.41 | 52186 (61.26) | 868 (44.33) | 53054 (60.88) | 16.36 |
| **1 CC** | 11388 (29.87) | 442 (32.45) | 11830 (29.96) | 37.36 | 11776 (25.02) | 193 (32.38) | 11969 (25.11) | 16.12 | 23164 (27.19) | 635 (32.431) | 23799 (27.31) | 26.68 |
| **2 CC** | 4165 (10.93) | 217 (15.93) | 4382 (11.098) | 49.52 | 2912 (6.19) | 71 (11.91) | 2983 (6.26) | 23.80 | 7077 (8.31) | 288 (14.71) | 7365 (8.45) | 39.1 |
| **3 CC** | 1273 (3.34) | 86 (6.31) | 1359 (3.44) | 63.28 | 655 (1.39) | 22 (3.69) | 677 (1.42) | 32.50 | 1928 (2.26) | 108 (5.52) | 2036 (2.34) | 53.05 |
| **≥ 4 CC** | 572 (1.50) | 48 (3.52) | 620 (1.57) | 77.42 | 258 (0.55) | 11 (1.85) | 269 (0.56) | 40.89 | 830 (0.97) | 59 (3.01) | 889 (1.02) | 66.37 |
| **p-value** |  |  |  | p<0.0001 |  |  |  | p<0.0001 |  |  |  | p<0.0001 |
| **Age (years) means (SD)** | |  |  |  |  |  |  |  |  |  |  |  |
|  | 32.04 (8.58) | 37.75 (12.14) | 32.24 (16.81) |  | 29.01 (7.48) | 32.81 (9.23) | 29.06 (7.51) |  | 30.37 (8.13) | 36.25 (11.56) | 30.51 (8.27) |  |
| **Age groups (years)** |  |  |  |  |  |  |  |  |  |  |  |  |
| **≤ 39 years** | 30661 (80.44) | 810 (59.47) | 31471 (79.71) | 25.74 | 42113 (89.50) | 450 (75.5) | 42563 (89.32) | 10.57 | 72774 (85.44) | 1260 (85.44) | 74034 (64.35) | 17.02 |
| **40-59 years** | 7268 (19.04) | 468 (34.36) | 7736 (19.6) | 60.5 | 4902 (10.42) | 142 (23.823) | 5044 (10.59) | 28.15 | 12170 (14.29) | 610 (14.29) | 12780 (31.15) | 47.73 |
| **≥ 60 years** | 189 (0.50) | 84 (6.17) | 273 (0.69) | 307.7 | 41 (0.08) | 4 (0.67) | 45 (0.09) | 88.89 | 230 (0.27) | 88 (0.27) | 318 (4.49) | 276.73 |
| **p-value** |  |  |  | p<0.0001 |  |  |  | p<0.0001 |  |  |  | p<0.0001 |
| **Marital status** |  |  |  |  |  |  |  |  |  |  |  |  |
| **Single** | 17422 (47.05) | 519 (39.77) | 17941 (46.8) | 28.93 | 27231 (59.61) | 308 (53.94) | 27539 (59.54) | 11.18 | 44653 (53.99) | 827 (44.08) | 45480 (53.77) | 18.18 |
| **Living with partner** | 1933 (5.22) | 56 (4.29) | 1989 (5.19) | 28.15 | 2338 (5.12) | 28 (4.9) | 2366 (5.12) | 11.83 | 4271 (5.16) | 84 (4.48) | 4355 (5.15) | 19.29 |
| **Married** | 17673 (47.73) | 730 (55.94) | 18403 (48.01) | 39.67 | 16112 (35.27) | 235 (41.16) | 16347 (35.34) | 14.38 | 33785 (40.85) | 965 (51.44) | 34750 (41.08) | 27.77 |
| **p-value** |  |  |  | p<0.0001 |  |  |  | p=0.0130 |  |  |  | p<0.0001 |
| **Geographic Regions** | | |  |  |  |  |  |  |  |  |  |  |
| **Bangkok** | 5522 (14.62) | 193 (14.31) | 5715 (14.61) | 33.77 | 9028 (19.32) | 120 (20.27) | 9148 (19.41) | 13.12 | 14550 (17.22) | 313 (16.13) | 14863 (17.19) | 21.06 |
| **Central** | 8582 (22.72) | 285 (21.13) | 8867 (22.66) | 32.14 | 12145 (25.99) | 154 (26.01) | 12299 (26.09) | 12.52 | 20727 (24.53) | 439 (22.62) | 21166 (24.49) | 20.74 |
| **North** | 7391 (19.57) | 268 (19.87) | 7659 (19.58) | 34.99 | 7987 (17.09) | 108 (18.24) | 8095 (17.17) | 13.34 | 15378 (18.20) | 376 (19.37) | 15754 (18.22) | 23.87 |
| **Northeast** | 9207 (24.37) | 347 (25.72) | 9554 (24.42) | 36.32 | 8382 (17.94) | 102 (17.23) | 8484 (18.00) | 12.02 | 17589 (20.82) | 449 (23.13) | 18038 (20.87) | 24.89 |
| **East** | 2312 (6.12) | 85 (6.30) | 2397 (6.13) | 35.46 | 2898 (6.20) | 32 (5.41) | 2,930 (6.22) | 10.92 | 5,210 (6.17) | 117 (6.03) | 5,327 (6.16) | 21.96 |
| **South** | 4,762 (12.61) | 171 (12.68) | 4,933 (12.61) | 34.66 | 6,285 (13.45) | 76 (12.84) | 6,361 (13.49) | 11.95 | 11,047 (13.07) | 247 (12.73) | 11,294 (13.07) | 21.87 |
| **p-value** |  |  |  | p=0.7540 |  |  |  | p=0.8950 |  |  |  | p=0.0580 |
| **Life course residence**** | |  |  |  |  |  |  |  |  |  |  |  |
| **Rural-rural (RR)** | 17,012 (45.27) | 595 (45.04) | 17607 (45.26) | 33.79 | 19,899 (42.75) | 238 (40.55) | 20137 (42.72) | 11.82 | 36,911 (43.88) | 833 (43.69) | 37744 (43.87) | 22.07 |
| **Rural-urban (RU)** | 12,227 (32.53) | 392 (29.67) | 12619 (32.44) | 31.06 | 14,657 (31.49) | 157 (26.75) | 14814 (31.43) | 10.6 | 26,884 (31.95) | 549 (28.77) | 27433 (31.89) | 20.01 |
| **Urban-rural (UR)** | 1,621 (4.31) | 78 (5.90) | 1699 (4.37) | 45.91 | 1,981 (4.26) | 27 (4.60) | 2008 (4.26) | 13.45 | 3,602 (4.28) | 105 (5.50) | 3707 (4.31) | 28.32 |
| **Urban-urban (UU)** | 6,722 (17.89) | 256 (19.38) | 6978 (17.94) | 36.67 | 10,008 (21.50) | 165 (28.11) | 10173 (21.58) | 16.22 | 16,730 (19.89) | 421 (22.06) | 17151 (19.93) | 24.55 |
| **p-value** |  |  |  | p=0.0010 |  |  |  | p=0.0070 |  |  |  | p=0.001 |
| **Education level** |  |  |  |  |  |  |  |  |  |  |  |  |
| **Junior school** | 1845 (4.85) | 126 (9.29) | 1971 (5.00) | 63.93 | 1049 (2.24) | 19 (3.19) | 1068 (2.25) | 17.79 | 2894 (3.41) | 145 (7.43) | 3039 (3.50) | 47.71 |
| **High school** | 19115 (50.27) | 664 (48.93) | 19779 (50.22) | 33.57 | 19365 (41.27) | 270 (45.38) | 19635 (41.32) | 13.75 | 38480 (45.3) | 934 (47.85) | 39414 (45.35) | 23.7 |
| **Diploma** | 8609 (22.64) | 294 (21.67) | 8903 (22.61) | 33.02 | 14384 (30.65) | 181 (30.42) | 14565 (30.655) | 12.43 | 22993 (27.07) | 475 (24.34) | 23468 (27.00) | 20.24 |
| **University** | 8459 (22.24) | 273 (20.12) | 8732 (22.17) | 31.26 | 12128 (25.84) | 125 (21.01) | 12253 (25.78) | 10.2 | 20587 (24.22) | 398 (20.39) | 20985 (24.15) | 18.97 |
| **p-value** |  |  |  | p<0.0001 |  |  |  | p=0.0170 |  |  |  | p<0.0001 |
| **Personal monthly income (baht)** | |  |  |  |  |  |  |  |  |  |  |  |
| **≤ 7000 baht** | 13166 (35.39) | 443 (34.05) | 13609 (35.35) | 32.55 | 21775 (47.47) | 251 (43.2) | 22026 (47.41) | 11.40 | 34941 (42.06) | 694 (36.88) | 35635 (41.94) | 19.48 |
| **7001-10000 baht** | 8539 (22.95) | 282 (21.68) | 8821 (22.91) | 31.97 | 10860 (23.67) | 117 (20.14) | 10977 (23.63) | 10.66 | 19399 (23.35) | 399 (21.20) | 19798 (23.30) | 20.15 |
| **10001-20000 baht** | 10561 (28.39) | 360 (27.67) | 10921 (28.36) | 32.96 | 9503 (20.72) | 145 (24.96) | 9648 (20.77) | 15.03 | 20064 (24.15) | 505 (26.83) | 20569 (24.21) | 24.55 |
| **≥ 20001 baht** | 4936 (13.27) | 216 (16.60) | 5152 (13.38) | 41.93 | 3735 (8.14) | 68 (11.70) | 3803 (8.19) | 17.88 | 8671 (10.44) | 284 (15.09) | 8955 (10.54) | 31.71 |
| **p-value** |  |  |  | p=0.0010 |  |  |  | p<0.0001 |  |  |  | p<0.0001 |

CMR per 1000*: 15-year cumulative mortality probability per 1000. Life course residence**: Life course residence was categorized in terms of rural (R) or urban (U) residential residence, when aged 10–12 years old and again in 2005, creating four groups: lifelong ruralites (Rural-rural, RR), urbanizers (Rural-urban, RU), de-urbanizers (Urban-rural, UR) and urbanites (Urban-urban, UU) (16, 19, 21, 22).

**Supplementary Table 2: Univariate hazard ratios (HRs) according to multimorbidity, number of chronic conditions (CC) and risk factors (Thailand, 2005 for baseline characteristics and 2019 for all-cause mortality).**

| **Participants** |  |  | |  | |  | | |  | | |  |  | | |  |  |  |
| --- | --- | --- | --- | --- | --- | --- | --- | --- | --- | --- | --- | --- | --- | --- | --- | --- | --- | --- |
|  | **Male** |  | |  | | **Female** | | |  | | |  | **Total** | | |  |  |  |
|  | **HR** | **(95 % CI)** | | | | **HR** | | | **(95 % CI)** | | | | **HR** | | | **(95 % CI)** | |  |
| **Two or more chronic conditions** | | | | | | | | | | | | | | | | | |  |
| **Multimorbidity** |  | | | | | | | | | | | | | | | | |  |
| **No** | 1.00 |  | | | | 1.00 | | |  | | | | 1.00 | | |  | |  |
| **Yes** | 1.83 | (1.62, | 2.06) | | | 2.37 | | | (1.92, | | 2.93) | | 2.29 | | | (2.06, | 2.54) |  |
| **Number of chronic conditions (CC)** | | | | | | | | | | | | | | | | | |  |
| **0 CC** | 1.00 |  | |  | | 1.00 | | |  | | |  | 1.00 | | |  |  |  |
| **1 CC** | 1.41 | (1.24, | | 1.59) | | 1.72 | | | (1.43, | | | 2.06) | 1.64 | | | (1.48, | 1.82) |  |
| **2 CC** | 1.87 | (1.60, | | 2.19) | | 2.55 | | | (1.97, | | | 3.30) | 2.41 | | | (2.11, | 2.76) |  |
| **3 CC** | 2.40 | (1.92, | | 3.01) | | 3.49 | | | (2.26, | | | 5.38) | 3.29 | | | (2.70, | 4.02) |  |
| **≥ 4 CC** | 2.97 | (2.21, | | 3.98) | | 4.38 | | | (2.40, | | | 8.00) | 4.15 | | | (3.19, | 5.40) |  |
| **p-trend** | **0.0001** |  | |  | | **0.0001** | | |  | | |  | **0.0001** | | |  |  |  |
| **Socio-demographic factors** | |  | |  | |  | | |  | | |  |  | | |  |  |  |
| **Gender** | 1.00 |  | |  | | 0.36 | | | (0.33, | | | 0.40) |  | | |  |  |  |
| **Age (years)** |  | | | | | | | | | | | | | | | | |  |
| **≤ 39 years** | 1.00 |  | | |  | | 1.00 |  | |  | | | | 1.00 |  | |  |  |
| **40-59 years** | 2.39 | (2.13, | | 2.67) | | 2.68 | | | (2.22, | | | 3.24) | 2.84 | | | (2.58, | 3.13) |  |
| **≥ 60 years** | 13.79 | (11.02, | | 17.27) | | 8.61 | | | (3.22, | | | 23.05) | 18.47 | | | (14.88, | 22.93) |  |
| **p-trend** | 0.1972 |  | |  | | 0.1220 | | |  | | |  | 0.2520 | | |  |  |  |
| **Marital status** |  | | | | | | | | | | | | | | | | |  |
| **Single** | 1.00 |  | |  | | 1.00 | | |  | | |  | 1.00 | | |  |  |  |
| **Living with partner** | 0.97 | (0.74, | | 1.28) | | 1.06 | | | (0.72, | | | 1.56) | 1.06 | | | (0.85, | 1.33) |  |
| **Married** | 1.38 | (1.23, | | 1.54) | | 1.29 | | | (1.09, | | | 1.52) | 1.53 | | | (1.40, | 1.68) |  |
| **Geographic regions** |  | | | | | | | | | | | | | | | | |  |
| **Bangkok** | 1.00 |  | |  | | 1.00 | | |  | | |  | 1.00 | | |  |  |  |
| **Central** | 0.95 | (0.79, | | 1.14) | | 0.95 | | | (0.75, | | | 1.21) | 0.98 | | | (0.85, | 1.14) |  |
| **North** | 1.04 | (0.86, | | 1.25) | | 1.02 | | | (0.78, | | | 1.32) | 1.13 | | | (0.98, | 1.32) |  |
| **Northeast** | 1.08 | (0.90, | | 1.28) | | 0.92 | | | (0.70, | | | 1.19) | 1.18 | | | (1.02, | 1.37) |  |
| **East** | 1.05 | (0.81, | | 1.36) | | 0.83 | | | (0.56, | | | 1.23) | 1.04 | | | (0.84, | 1.29) |  |
| **South** | 1.03 | (0.84, | | 1.26) | | 0.91 | | | (0.68, | | | 1.21) | 1.04 | | | (0.88, | 1.23) |  |
| **Life course residence*** | | | | | | | | | | | | | | | | | |  |
| **Rural-rural (RR)** | 1.00 |  | |  | | 1.00 | | |  | | |  | 1.00 | | |  |  |  |
| **Rural-urban (RU)** | 0.92 | (0.81, | | 1.04) | | 0.90 | | | (0.73, | | | 1.10) | 0.91 | | | (0.81, | 1.01) |  |
| **Urban-rural (UR)** | 1.37 | (1.08, | | 1.73) | | 1.14 | | | (0.76, | | | 1.70) | 1.29 | | | (1.05, | 1.58) |  |
| **Urban-urban (UU)** | 1.08 | (0.94, | | 1.26) | | 1.38 | | | (1.13, | | | 1.68) | 1.11 | | | (0.99, | 1.25) |  |
| **Education level** |  | | | | | | | | | | | | | | | | |  |
| **Junior school** | 1.00 |  | |  | | 1.00 | | |  | | |  | 1.00 | | |  |  |  |
| **High school** | 0.52 | (0.43, | | 0.63) | | 0.77 | | | (0.49, | | | 1.23) | 0.49 | | | (0.41, | 0.58) |  |
| **Diploma** | 0.51 | (0.41, | | 0.63) | | 0.70 | | | (0.44, | | | 1.12) | 0.42 | | | (0.35, | 0.50) |  |
| **University** | 0.48 | (0.39, | | 0.59) | | 0.57 | | | (0.35, | | | 0.93) | 0.39 | | | (0.32, | 0.47) |  |
| **p-trend** | 0.1891 |  | |  | | **0.0243** | | |  | | |  | 0.1436 | | |  |  |  |
| **Personal monthly income (baht)** | | | | | | | | | | | | | | | | | |  |
| **≤ 7000 baht** | 1.00 |  | |  | | 1.00 | | |  | | |  | 1.00 | | |  |  |  |
| **7001-10000 baht** | 0.98 | (0.84, | | 1.14) | | 0.93 | | | (0.75, | | | 1.16) | 1.03 | | | (0.91, | 1.17) |  |
| **10001-20000 baht** | 1.01 | (0.88, | | 1.16) | | 1.32 | | | (1.08, | | | 1.62) | 1.26 | | | (1.13, | 1.42) |  |
| **≥ 20001 baht** | 1.29 | (1.10, | | 1.52) | | 1.57 | | | (1.20, | | | 2.05) | 1.64 | | | (1.42, | 1.88) |  |
| **p-trend** | **0.0380** |  | |  | | 0.0650 | | |  | | |  | **0.0077** | | |  |  |  |
| **Health behaviours/ Personal lifestyles** | | | | | |  | | |  | | |  |  | | |  |  |  |
| **Diet consumptions** |  |  | |  | |  | | |  | | |  |  | | |  |  |  |
| **Deep fried food** |  | | | | | | | | | | | | | | | | |  |
| **Never** | 1.00 |  | |  | | 1.00 | | |  | | |  | 1.00 | | |  |  |  |
| **1-3 times/month** | 0.66 | (0.49, | | 0.89) | | 0.63 | | | (0.41, | | | 0.98) | 0.63 | | | (0.50, | 0.81) |  |
| **1-2 times/week** | 0.54 | (0.41, | | 0.71) | | 0.66 | | | (0.43, | | | 1.00) | 0.58 | | | (0.46, | 0.73) |  |
| **3-6 times/week** | 0.53 | (0.40, | | 0.70) | | 0.54 | | | (0.35, | | | 0.82) | 0.56 | | | (0.44, | 0.70) |  |
| **Daily or more** | 0.52 | (0.39, | | 0.70) | | 0.50 | | | (0.32, | | | 0.78) | 0.53 | | | (0.41, | 0.68) |  |
| **p-trend** | 0.0716 |  | |  | | 0.0531 | | |  | | |  | 0.0854 | | |  |  |  |
| **Instant food** |  | | | | | | | | | | | | | | | | |  |
| **Never** | 1.00 |  | |  | | 1.00 | | |  | | |  | 1.00 | | |  |  |  |
| **1-3 times/month** | 0.68 | (0.59, | | 0.78) | | 0.74 | | | (0.60, | | | 0.92) | 0.67 | | | (0.59, | 0.75) |  |
| **1-2 times/week** | 0.67 | (0.58, | | 0.78) | | 0.69 | | | (0.54, | | | 0.87) | 0.66 | | | (0.58, | 0.75) |  |
| **3-6 times/week** | 0.70 | (0.58, | | 0.83) | | 0.74 | | | (0.56, | | | 0.98) | 0.70 | | | (0.60, | 0.81) |  |
| **Daily or more** | 0.57 | (0.39, | | 0.83) | | 0.65 | | | (0.38, | | | 1.11) | 0.56 | | | (0.41, | 0.77) |  |
| **p-trend** | 0.0910 |  | |  | | 0.0967 | | |  | | |  | 0.0975 | | |  |  |  |
| **Soft drink** |  | | | | | | | | | | | | | | | | |  |
| **Never** | 1.00 |  | |  | | 1.00 | | |  | | |  | 1.00 | | |  |  |  |
| **1-3 times/month** | 0.81 | (0.70, | | 0.94) | | 0.78 | | | (0.63, | | | 0.97) | 0.88 | | | (0.77, | 0.99) |  |
| **1-2 times/week** | 0.73 | (0.63, | | 0.85) | | 0.76 | | | (0.60, | | | 0.96) | 0.86 | | | (0.76, | 0.98) |  |
| **3-6 times/week** | 0.73 | (0.62, | | 0.87) | | 0.97 | | | (0.76, | | | 1.24) | 0.95 | | | (0.83, | 1.09) |  |
| **Daily or more** | 0.72 | (0.57, | | 0.91) | | 0.97 | | | (0.71, | | | 1.32) | 0.88 | | | (0.73, | 1.06) |  |
| **p-trend** | 0.0679 |  | |  | | 0.7935 | | |  | | |  | 0.4406 | | |  |  |  |
| **Soybean products** |  | | | | | | | | | | | | | | | | |  |
| **Never** | 1.00 |  | |  | | 1.00 | | |  | | |  | 1.00 | | |  |  |  |
| **1-3 times/month** | 0.83 | (0.71, | | 0.98) | | 0.80 | | | (0.59, | | | 1.08) | 0.80 | | | (0.69, | 0.92) |  |
| **1-2 times/week** | 0.89 | (0.76, | | 1.05) | | 1.02 | | | (0.77, | | | 1.35) | 0.85 | | | (0.74, | 0.98) |  |
| **3-6 times/week** | 0.94 | (0.80, | | 1.12) | | 1.06 | | | (0.80, | | | 1.40) | 0.85 | | | (0.73, | 0.98) |  |
| **Daily or more** | 1.03 | (0.83, | | 1.29) | | 1.07 | | | (0.79, | | | 1.46) | 0.81 | | | (0.68, | 0.96) |  |
| **p-trend** | 0.5643 |  | |  | | 0.3128 | | |  | | |  | 0.2364 | | |  |  |  |
| **Vegetable (serves/day)** | | | | | | | | | | | | | | | | | |  |
| **0-4** | 1.00 |  | |  | | 1.00 | | |  | | |  | 1.00 | | |  |  |  |
| **5-9** | 1.07 | (0.84, | | 1.37) | | 0.68 | | | (0.43, | | | 1.08) | 0.96 | | | (0.77, | 1.19) |  |
| **10-14** | 0.82 | (0.51, | | 1.33) | | 1.23 | | | (0.66, | | | 2.29) | 0.97 | | | (0.66, | 1.42) |  |
| **≥ 15** | 1.80 | (1.02, | | 3.18) | | 0.36 | | | (0.05, | | | 2.57) | 1.41 | | | (0.82, | 2.43) |  |
| **p-trend** | 0.2120 |  | |  | | 0.4047 | | |  | | |  | 0.1327 | | |  |  |  |
| **Fruit (serves/day)** |  | | | | | | | | | | | | | | | | |  |
| **0-4** | 1.00 |  | |  | | 1.00 | | |  | | |  | 1.00 | | |  |  |  |
| **5-9** | 0.95 | (0.80, | | 1.13) | | 0.90 | | | (0.71, | | | 1.14) | 0.86 | | | (0.75, | 0.99) |  |
| **10-14** | 1.04 | (0.80, | | 1.35) | | 0.77 | | | (0.51, | | | 1.16) | 0.89 | | | (0.71, | 1.11) |  |
| **≥ 15** | 1.42 | (0.93, | | 2.17) | | 0.67 | | | (0.30, | | | 1.51) | 1.07 | | | (0.74, | 1.56) |  |
| **p-trend** | 0.0841 |  | |  | | **0.0406** | | |  | | |  | 0.4517 | | |  |  |  |
| **Activity status** |  |  | |  | |  | | |  | | |  |  | | |  |  |  |
| **Weekly exercise (sessions)#** | | | | | | | | | | | | | | | | | |  |
| **0-7** | 1.00 |  | |  | | 1.00 | | |  | | |  | 1.00 | | |  |  |  |
| **8-14** | 0.89 | (0.78, | | 1.01) | | 0.99 | | | (0.82, | | | 1.20) | 1.08 | | | (0.97, | 1.20) |  |
| **≥ 15** | 0.95 | (0.84, | | 1.08) | | 1.16 | | | (0.92, | | | 1.45) | 1.32 | | | (1.18, | 1.47) |  |
| **p-trend** | 0.7514 |  | |  | | 0.2735 | | |  | | |  | 0.0877 | | |  |  |  |
| **Housework** |  | | | | | | | | | | | | | | | | |  |
| **Never** | 1.00 |  | |  | | 1.00 | | |  | | |  | 1.00 | | |  |  |  |
| **1-3 times/month** | 0.89 | (0.71, | | 1.12) | | 0.84 | | | (0.54, | | | 1.30) | 0.85 | | | (0.69, | 1.04) |  |
| **Once or twice/week** | 0.81 | (0.66, | | 1.00) | | 0.69 | | | (0.47, | | | 1.01) | 0.67 | | | (0.56, | 0.81) |  |
| **3-4 times/week** | 0.81 | (0.64, | | 1.02) | | 0.64 | | | (0.41, | | | 0.98) | 0.67 | | | (0.54, | 0.82) |  |
| **Every day or more** | 1.20 | (0.99, | | 1.46) | | 0.70 | | | (0.49, | | | 1.02) | 0.79 | | | (0.67, | 0.94) |  |
| **p-trend** | 0.6186 |  | |  | | 0.0665 | | |  | | |  | 0.2002 | | |  |  |  |
| **Sleeping time (hours)** |  | | | | | | | | | | | | | | | | |  |
| **≤ 6 hours** | 1.20 | (1.07, | | 1.35) | | 1.00 | | | (0.83, | | | 1.20) | 1.17 | | | (1.06, | 1.29) |  |
| **7-8 hours** | 1.00 |  | |  | | 1.00 | | |  | | |  | 1.00 | | |  |  |  |
| **≥ 9 hours** | 1.25 | (1.06, | | 1.47) | | 1.17 | | | (0.93, | | | 1.47) | 1.19 | | | (1.04, | 1.36) |  |
| **p-trend** | 0.8852 |  | |  | | 0.3176 | | |  | | |  | 0.9389 | | |  |  |  |
| **Sedentary time (hours)** |  | | | | | | | | | | | | | | | | |  |
| **≤ 7 hours** | 1.00 |  | |  | | 1.00 | | |  | | |  | 1.00 | | |  |  |  |
| **8-12 hours** | 0.88 | (0.78, | | 0.99) | | 1.19 | | | (0.99, | | | 1.45) | 0.90 | | | (0.81, | 1.00) |  |
| **≥ 13 hours** | 0.84 | (0.73, | | 0.97) | | 1.11 | | | (0.90, | | | 1.36) | 0.80 | | | (0.72, | 0.90) |  |
| **p-trend** | 0.1960 |  | |  | | 0.6231 | | |  | | |  | **0.0051** | | |  |  |  |
| **Smoking and Drinking status** | | | |  | |  | | |  | | |  |  | | |  |  |  |
| **Smoking** |  | | | | | | | | | | | | | | | | |  |
| **Never smoking** | 1.00 |  | |  | | 1.00 | | |  | | |  | 1.00 | | |  |  |  |
| **Smoking** | 1.79 | (1.59, | | 2.00) | | 1.50 | | | 1.11, | | | 2.01) | 2.58 | | | (2.36, | 2.82) |  |
| **Drinking** |  | | | | | | | | | | | | | | | | |  |
| **Never** | 1.00 |  | |  | | 1.00 | | |  | | |  | 1.00 | | |  |  |  |
| **Ex-drinker** | 1.12 | (0.94, | | 1.35) | | 1.16 | | | (0.97, | | | 1.37) | 0.81 | | | (0.72, | 0.91) |  |
| **Occasional or social** | 1.62 | (1.37, | | 1.90) | | 2.31 | | | (1.14, | | | 4.67) | 2.22 | | | (1.90, | 2.59) |  |
| **Current regular** | 2.03 | (1.76, | | 2.34) | | 1.61 | | | (1.22, | | | 2.13) | 2.02 | | | (1.78, | 2.29) |  |
| **p-trend** | **0.0232** |  | |  | | 0.3420 | | |  | | |  | 0.3420 | | |  |  |  |

p-trend **bolding**: The results of p-trend were statistical significance. Life course residence*: Life course residence was categorized in terms of rural (R) or urban (U) residential residence, when aged 10–12 years old and again in 2005, creating four groups: lifelong ruralites (Rural-rural, RR), urbanizers (Rural-urban, RU), de-urbanizers (Urban-rural, UR) and urbanites (Urban-urban, UU) (16, 19, 21, 22). Weekly exercise#: The Combined measure of weekly physical activities: “2 × vigorous exercises + 1 × moderate exercises + 1 × walking sessions per week”, which was on the basis of International Physical Activity Questionnaire and the Active Australia Survey (21, 22).
